# Li_2.9_Fe_0.9_Zr_0.1_Cl_6_ as Redox-Active Catholyte for Solid-State Li-Ion Batteries

**DOI:** 10.1021/acs.chemmater.4c01385

**Published:** 2024-10-07

**Authors:** Guangxing Zhang, Zhantao Liu, Yifan Ma, Jakub Pepas, Jianming Bai, Hui Zhong, Yuanzhi Tang, Hailong Chen

**Affiliations:** † The Woodruff School of Mechanical Engineering, Georgia Institute of Technology, Atlanta, Georgia 30332, United States; ‡ School of Materials Science and Engineering, Georgia Institute of Technology, Atlanta, Georgia 30332, United States; § National Synchrotron Light Source II, Brookhaven National Laboratory, Upton, New York 11973, United States; ∥ School of Earth and Atmospheric Sciences, Georgia Institute of Technology, Atlanta, Georgia 30332, United States

## Abstract

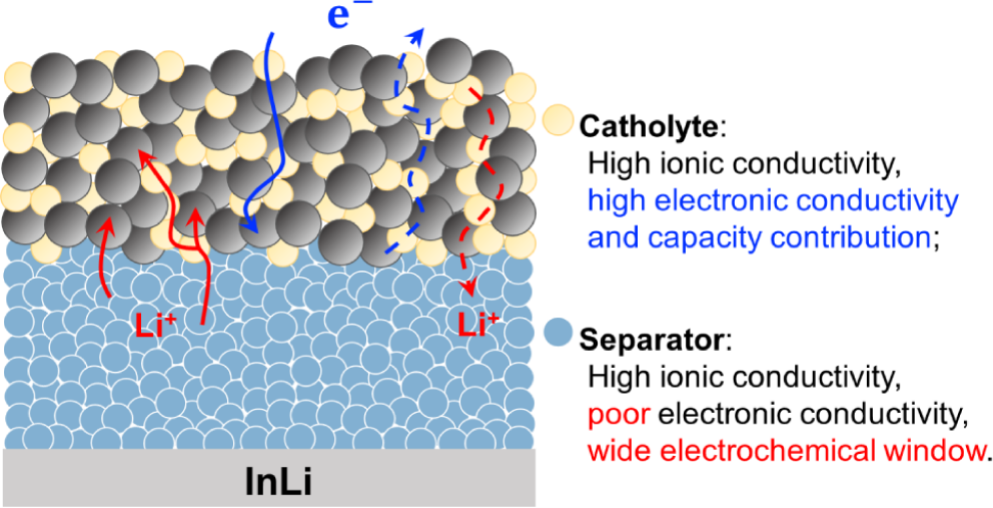

Solid electrolytes are one of the key challenges that hinder the commercialization of all-solid-state batteries. Most efforts have been made to advance the development of solid electrolytes as separators, while the development of catholytes, particularly redox-active catholytes, has been less extensively studied. The high loading of catholytes in composite cathodes, while facilitating ionic conduction, drastically decreases the energy density of the battery. Here, we report an alternative strategy to improve the energy density by using Li_2.9_Fe_0.9_Zr_0.1_Cl_6_ as a redox-active catholyte. With a composite cathode containing uncoated LiCoO_2_ and Li_2.9_Fe_0.9_Zr_0.1_Cl_6_, the solid-state cell not only shows excellent rate capability and stable long-term cycling, benefiting from the high ionic conductivity of Li_2.9_Fe_0.9_Zr_0.1_Cl_6_, but also shows a high cathode specific capacity of ∼153 mAh·g^–1^. This study broadens the chemical space of the materials design for lithium-ion conductors with redox-active elements (e.g., Fe, Ti, V, and Cr), offering new opportunities to reduce the cost and improve the energy density for all-solid-state batteries.

## 1 Introduction

By utilizing nonflammable solid electrolytes (SEs), all-solid-state batteries (ASSBs) greatly reduce the risk of thermal runaway and offer superior safety properties over conventional Li-ion batteries (LIBs).^1−4^ The use of SEs also potentially enables the safe implementation of Li-metal anodes, thus contributing to a substantial increase in energy density due to the high specific capacity of Li (3860 mAh·g^–1^). In all-solid-state cells, SEs are used both as the separator and as the ionic conducting additive mixed with cathode active materials (CAMs) in the composite cathode.^5,6^ The SEs used in the composite cathode are commonly referred to as catholytes. The weight ratio of catholyte in the composite cathode is typically 20–30%,^7,8^ occasionally reaching up to 50%,^9^ to ensure sufficient Li^+^ transport within the cathode. A large portion of SEs significantly lowers the energy density of ASSBs.^10^ To improve the energy density of ASSBs, a straightforward strategy is to decrease the weight ratio of SEs by either improving the intrinsic conductivity of the SEs^2^ or nanosizing the SEs to offer a better ionic conducting network.^4,11^ Yet, an alternative strategy is to develop redox-active catholytes, which not only function as the ionic conduction media but also contribute to the overall capacity.

In the past, most solid-state Li-ion conductors were developed primarily for use as the separator, which requires a wide electrochemical stability window,^12−15^ thus eliminating the use of many redox-active transition metals, such as Fe, Co, Ni, and so on. In many cases, expensive elements (e.g., Y^3+^, In^3+^, Sc^3+^, Nb^5+^, Ta^5+^, lanthanide ions, and Zr^4+^) are heavily used in these SEs,^16−19^ which substantially increases the cost of ASSBs and hinders their commercialization. However, for a redox-active catholyte, redox-active elements can and should be used to provide capacity, which greatly expands the chemical space of the SE design. Similarly, high electronic conductivity is unfavorable for separators,^20,21^ while is highly preferred for a redox-active catholyte in order to provide electron-conducting pathways.^5,22^ In addition, good deformability^23^ and outstanding high-voltage stability are desirable for catholytes. Considering all these desired properties, we found transition-metal-based halides promising, as many of them exhibit good ionic and electronic conductivities^24^ and good deformability.^25^

In this work, we report a series of Fe-based halide catholytes that exhibit good ionic conductivity of over 0.1 mS·cm^–1^, electronic conductivities as high as 10^–7^ S·cm^–1^, and high capacity of ∼70 mAh·g^–1^. When Li_2.9_Fe_0.9_Zr_0.1_Cl_6_ is used as a catholyte, the ASSB using a LiCoO_2_ (LCO) and Li_2.9_Fe_0.9_Zr_0.1_Cl_6_ composite cathode demonstrates a specific capacity of 152.8 mAh·g^–1^ at 0.1 C and shows excellent rate and cycling performance. These findings offer new opportunities to develop new catholytes and a new approach to promote the energy density of ASSBs.

## 2 Results and Discussion

Previously, LiFeCl_4_ in *P*2_1_/*c* structure was investigated as an ionic conductor but showed a low ionic conductivity below 10^–6^ S·cm^–1^ at 25 °C.^26^ More recently, reports show that Li_3_*MX*_6_ (*M* = metal, *X* = halogens) group halide electrolytes with *C*2/*m* structures have a three-dimensional Li^+^ diffusion network, where Li^+^ diffusion within ab-planes is preferable with a lower diffusion barrier than that along the *c*-axis.^27−29^ The bond valence site energy (BVSE) calculation^30,31^ also shows that *C*2/*m* Li_3_FeCl_6_ exhibits a lower Li-ion migration barrier compared to that of LiFeCl_4_ in *P*2_1_/*c* structure (Figure S1). It appears to be an attractive and reasonable design strategy to synthesize Fe-based halide compounds in *C*2/*m* structures in order to simultaneously achieve a high ionic conductivity and high capacity.

**Figure 1:**
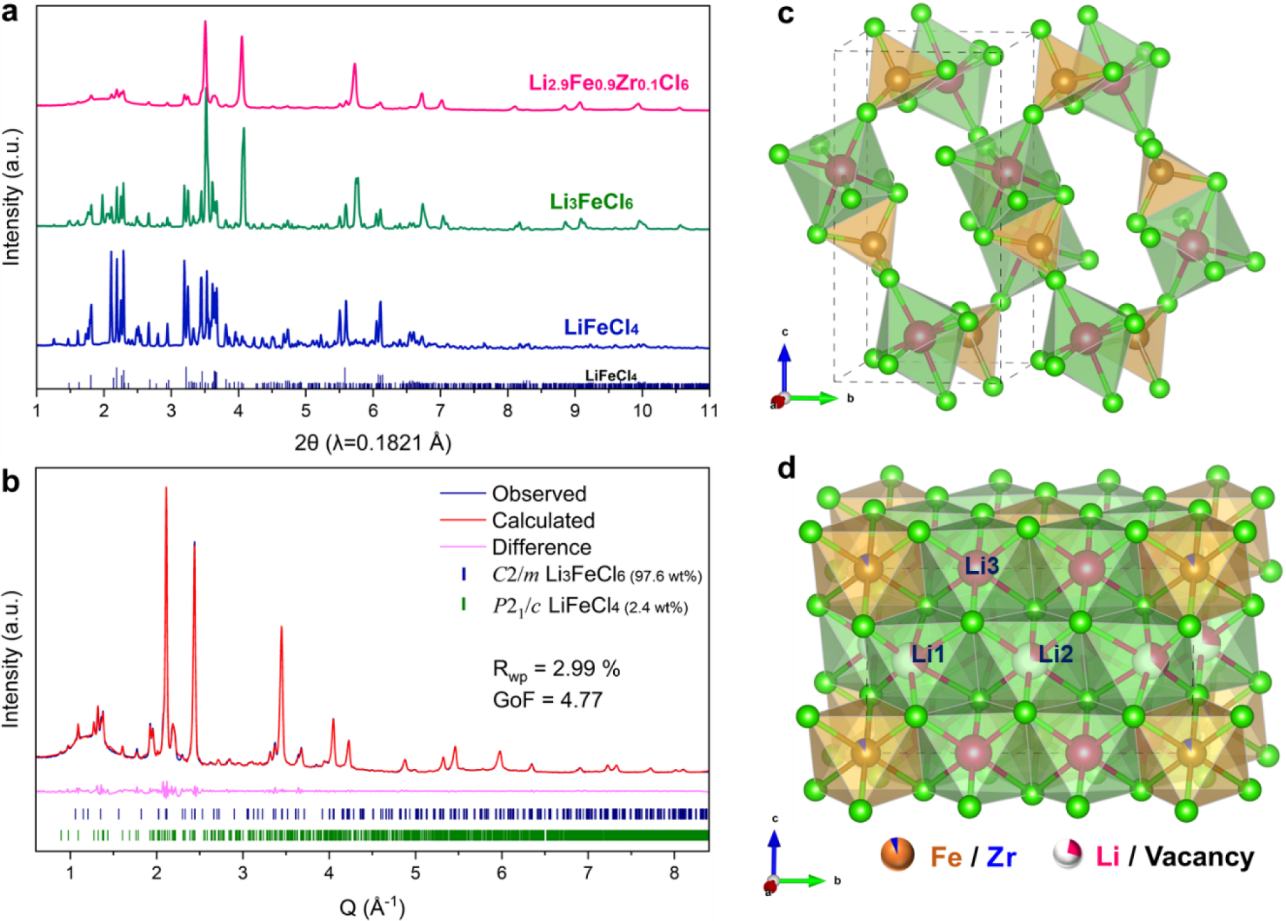
(a) XRD patterns of mechanochemically synthesized LiFeCl_4_, Li_3_FeCl_6_, and Li_2.9_Fe_0.9_Zr_0.1_Cl_6_. (b) Synchrotron X-ray diffraction pattern of Li_2.9_Fe_0.9_Zr_0.1_Cl_6_ and the corresponding Rietveld refinement. (c, d) Crystal structures of LiFeCl_4_ (*P*2_1_/*c*) and Li_2.9_Fe_0.9_Zr_0.1_Cl_6_ (*C*2/*m*) projected along the *a*-axis, respectively. Fe, Zr, Li, and Cl are represented by brown, blue, pink, and green spheres, respectively. The illustrative structures are visualized using VESTA.^32^

A series of Fe-based halide compounds LiFeCl_4_, Li_3_FeCl_6_, and Li_2.9_Fe_0.9_Zr_0.1_Cl_6_ were synthesized by mechanochemical method (see Section 4 for details) and their X-ray diffraction (XRD) patterns are shown in Figure 1a. The XRD pattern of as-milled LiFeCl_4_ can be well indexed to a monoclinic *P*2_1_/*c* space group, isostructural to LiAlCl_4_.^33^ The Fe atoms occupy the tetrahedral sites, and Li atoms reside at the octahedral sites, as shown in Figure 1c. Li_3_FeCl_6_ is predominantly crystallized in a *C*2/*m* phase with a small amount of impurity in a *P*2_1_/*c* phase. Based on the Rietveld refinement results in Figure S2, the proportion of *P*2_1_/*c* phase in the Li_3_FeCl_6_ sample is 13.7 wt %. Further increasing the ratio of LiCl to FeCl_3_ in the starting materials does not yield a pure *C*2/*m* structure. For example, starting materials with a nominal composition of Li_4_FeCl_7_ also yield a mixture of *P*2_1_/*c* and *C*2/*m* phases (Figure S3). Based on the hypothesis that using a cation with a bigger ionic radius to replace Fe^3+^ may drive the transition metal cations toward the dominance of octahedral sites and the observation that Zr^4+^ prefers to occupy octahedral sites in Zr-based halide electrolyte,^7,34^ we chose to dope 10% Zr^4+^ into the LiCl-FeCl_3_ starting materials, which successfully yielded a Li_2.9_Fe_0.9_Zr_0.1_Cl_6_ sample with predominant *C*2/*m* phase and a much-reduced *P*2_1_/*c* phased impurity fraction of 2.4 wt %. The schematic structure of Li_2.9_Fe_0.9_Zr_0.1_Cl_6_ with an *fcc* anion sublattice is illustrated in Figure 1d, drawn based on the Rietveld refinement results. After substituting Fe^3+^ with Zr^4+^, a slight increase of refined lattice parameters *a* and *b* for the *C*2/*m* phase can be observed in Tables S1 and S2, which can be attributed to the larger ion size of Zr^4+^ (72 pm) compared to that of Fe^3+^ (64.5 pm). The lattice expansion indicates the formation of the solid-solution phase, in which Zr^4+^ takes the Fe^3+^ sites. Further increasing the doping level of Zr, for example, to 30%, yields a pure *C*2/*m* phase as shown in Figure S4a. The corresponding Rietveld refinement result of Li_2.7_Fe_0.7_Zr_0.3_Cl_6_ is demonstrated in Figure S6. As the Zr content increases from 0.1 to 0.3 per formula unit, Bragg peaks gradually shift toward lower angles, indicating the expanded unit cell and further demonstrating the formation of the solid-solution phases (Figure S4b).

**Figure 2:**
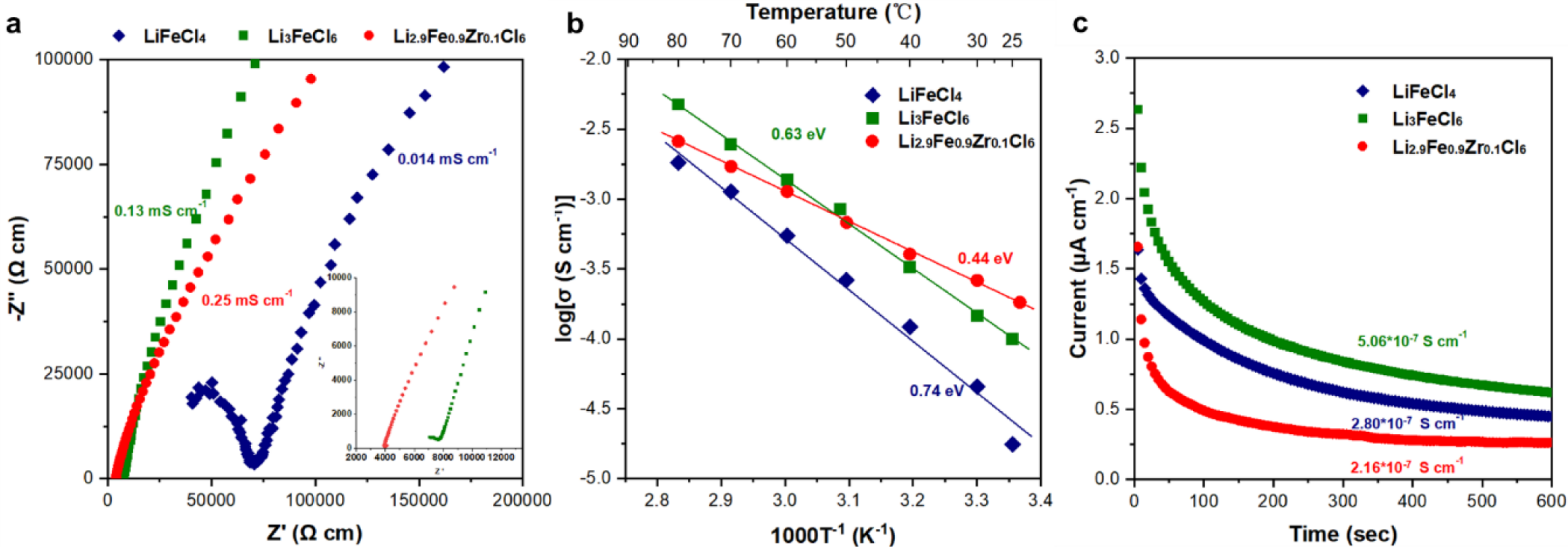
(a) Nyquist plots of the as-milled LiFeCl_4_, Li_3_FeCl_6_, and Li_2.9_Fe_0.9_Zr_0.1_Cl_6_ at 25 °C. (b) Arrhenius plots of all of the as-milled samples. (c) The transient current behavior under an applied 0.1 V DC bias on all of the samples.

Li_3_FeCl_6_, with a main phase of *C*2/*m*, demonstrates a good ionic conductivity of 0.13 mS·cm^–1^, which is attributed to the existence of 3D Li^+^ diffusion pathways. As a comparison, LiFeCl_4_ in the *P*2_1_/*c* structure shows an ionic conductivity of 0.014 mS·cm^–1^ at 25 °C, which is one order magnitude lower than that of Li_3_FeCl_6_. The ionic conductivities of Li_3-*x*_Fe_1–*x*_Zr_*x*_Cl_6_ (*x* = 0.1, 0.2, and 0.3) samples were measured via electrochemical impedance spectroscopy (EIS) as well. The introduction of Zr^4+^ at Fe^3+^ sites creates Li vacancies at the Li sites, which promotes the conductivity of Li^+^, similar to what was observed in aliovalent-doped SEs.^35−39^ Li_2.9_Fe_0.9_Zr_0.1_Cl_6_ exhibits an improved ionic conductivity of 0.25 mS·cm^–1^. With increased Zr doping levels, the ionic conductivity is further improved. Li_2.7_Fe_0.7_Zr_0.3_Cl_6_ shows an ionic conductivity of 0.37 mS·cm^–1^ (Figure S7b). Arrhenius plots of the conductivities of these samples are presented in Figure 2b. The activation energy of Li_2.9_Fe_0.9_Zr_0.1_Cl_6_ (0.44 eV) is found to be lower than those of LiFeCl_4_ (0.74 eV) and Li_3_FeCl_6_ (0.63 eV). The electronic conductivities of LiFeCl_4_, Li_3_FeCl_6_, and Li_2.9_Fe_0.9_Zr_0.1_Cl_6_ measured using a direct current (DC) polarization method are 2.80 × 10^–7^ S·cm^–1^, 5.06 × 10^–7^ S·cm^–1^ and 2.16 × 10^–7^ S·cm^–1^, respectively (Figure 2c). The electronic conductivities of Li_3-*x*_Fe_1–*x*_Zr_*x*_Cl_6_ compounds were also measured and shown in Figure S7d. With an increase in the Zr doping level, the electronic conductivity decreases. However, the increased doping level of electrochemical inactive Zr^4+^ lowers the specific capacity, which is not favorable for a catholyte. As the ionic conductivity of Li_2.9_Fe_0.9_Zr_0.1_Cl_6_ is sufficiently high, comparable to that of ball-milled Li_3_YCl_6_,^6,21^ a popularly used SE,^40,41^ and its specific capacity is sufficiently good, we chose to test the properties of Li_2.9_Fe_0.9_Zr_0.1_Cl_6_ as a catholyte. As a comparison, the performance of undoped Li_3_FeCl_6_ was measured to investigate the impact of Zr doping.

**Figure 3:**
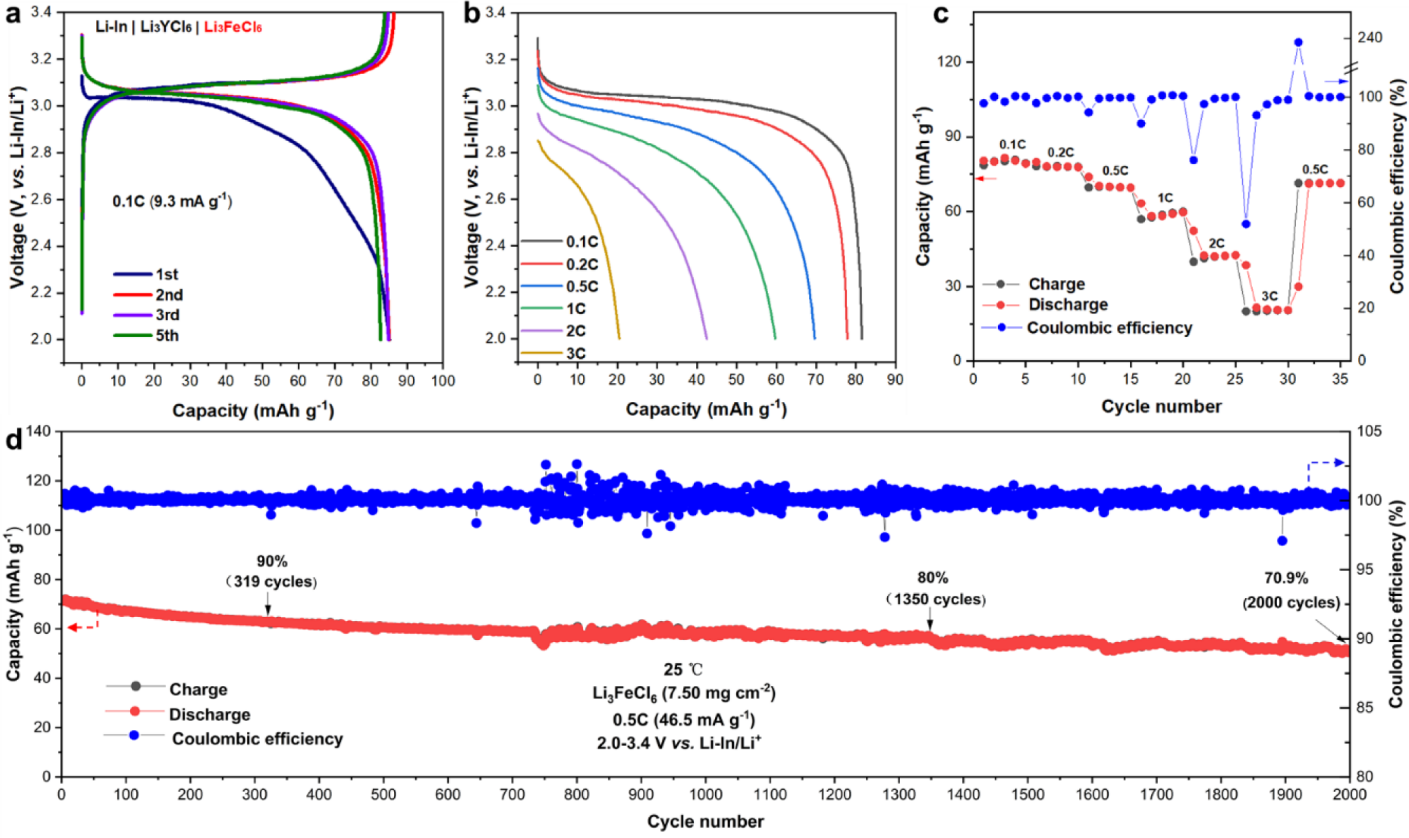
Electrochemical performance of the Li–In | Li_3_YCl_6_ | Li_3_FeCl_6_ cell at 25 °C. (a) The discharge–charge curves under 0.1 C (the theoretical capacity is 93 mAh·g^–1^). (b, c) Initial discharge curves at various rates and corresponding rate performances at 0.1, 0.2, 0.5, 1, 2, and 3 C. (d) Cycling performance and Coulombic efficiency under 0.5 C. All the cells were tested at 2.0–3.4 V vs Li–In/Li^+^.

To verify the feasibility of using Li_3_FeCl_6_ and Li_2.9_Fe_0.9_Zr_0.1_Cl_6_ as catholytes, we first investigated their cycling performance as the sole cathode material. For Li_3_FeCl_6_ cell tests, Li_3_YCl_6_ was used as the separator and Li–In alloy was used as the anode. The composite cathode contained 95 wt % of Li_3_FeCl_6_, 5 wt % of carbon nanofibers, and no other SEs. The voltage profiles of the Li–In | Li_3_YCl_6_ | Li_3_FeCl_6_ cell cycled between 2.0 and 3.4 V (vs Li–In/Li^+^) under 0.1 C (1 C = 93 mA·g^–1^) are shown in Figure 3a. The electrochemical reaction of Li_3_FeCl_6_ upon cycling is as follows, where the redox Fe^3+^/Fe^2+^ is utilized.
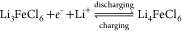


The major voltage plateau is between 3.0 and 3.1 V (i.e., 3.62–3.72 V vs Li^+^/Li). The cell shows an initial discharge capacity of 85.2 mAh·g^–1^ (91.6% of the theoretical capacity) and an excellent initial Coulombic efficiency of 99.5%. Even without additional SEs in the cathode, the Li–In | Li_3_YCl_6_ | Li_3_FeCl_6_ cells show a very good rate capability, as demonstrated in Figure 3b and 3c. Discharge capacities of 81.5, 77.8, 69.6, 59.7, 42.5, and 20.5 mAh·g^–1^ are achieved at room temperature under the rates of 0.1, 0.2, 0.5, 1, 2, and 3 C, respectively, corresponding to 87.6%, 83.7%, 74.8%, 64.2%, 45.7%, and 22.0% of the theoretical capacity, respectively. The cells also show excellent long-term cycling stability under 0.5 C at 25 °C as shown in Figure 3d with a 70.9% capacity retention after 2000 cycles and an average Coulombic efficiency of 99.98%.

Cyclic voltammetry (CV) tests were performed to investigate the characteristics of the Fe^2+^/Fe^3+^ redox couple and the electrochemical stability window using an all-solid-state Li–In | Li_3_YCl_6_ | Li_3_FeCl_6_ cell. As illustrated in Figure S8a, the oxidation of Fe^2+^ to Fe^3+^ emerges at ∼3.2 V vs. Li–In/Li^+^. As the scan proceeds, oxidation of Cl begins at ∼3.67 V vs Li–In/Li^+^. The peak corresponding to Cl oxidation diminishes after the fifth cycle, implying the formation of a passivation layer. When the cell was further cycled between 2.0 and 3.7 V vs Li–In/Li^+^, the peak positions and intensities of Fe^2+^/Fe^3+^ redox at different cycles remained unchanged, indicating the good reversibility and stability of the redox process (Figure S8b). Based on the CV scans, Fe-based halides demonstrate a reversible redox process and good stability when cycled between 2.0 and 3.7 V vs Li–In/Li^+^.

**Figure 4:**
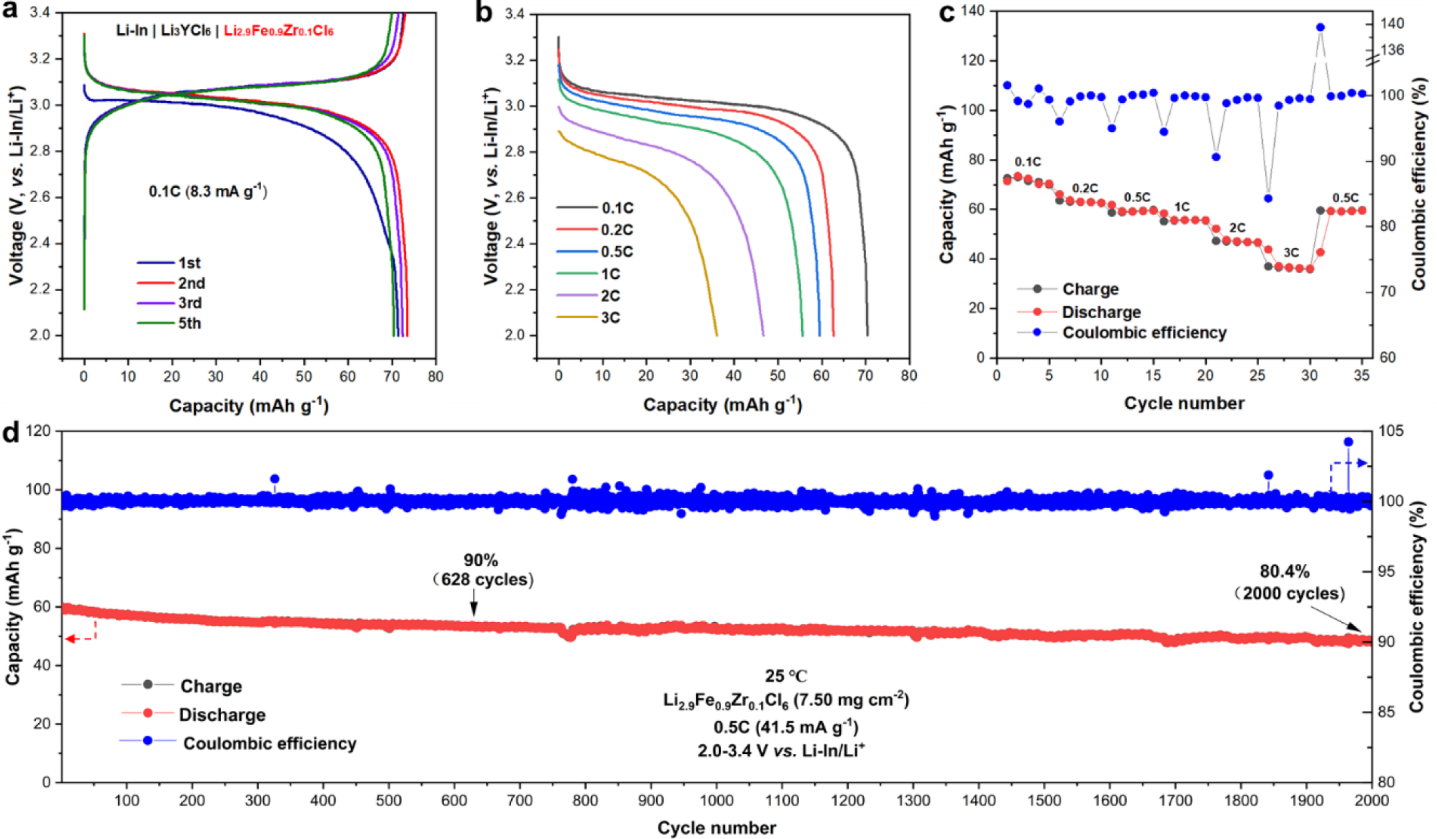
Electrochemical performance of the Li–In | Li_3_YCl_6_ | Li_2.9_Fe_0.9_Zr_0.1_Cl_6_ cell at 25 °C. (a) The discharge–charge curves under 0.1 C (the theoretical capacity is 83 mAh·g^–1^). (b, c) Initial discharge curves at various rates and corresponding rate performances at 0.1, 0.2, 0.5, 1, 2, and 3 C. (d) Cycling performance and Coulombic efficiency under 0.5 C. All the cells were tested at 2.0–3.4 V vs Li–In/Li^+^.

Li_2.9_Fe_0.9_Zr_0.1_Cl_6_ was also tested with the same configuration as that for Li_3_FeCl_6_. As shown in Figure 4a, the initial discharge capacity is 71.4 mAh·g^–1^, which is 86% of the theoretical capacity. The voltage plateau is the same as that of Li_3_FeCl_6_, from 2.8 to 3.0 V. This lower capacity than that of Li_3_FeCl_6_ is expected, resulting from the doping of the redox-nonactive Zr^4+^ cation. Benefiting from the higher ionic conductivity of Li_2.9_Fe_0.9_Zr_0.1_Cl_6_ than Li_3_FeCl_6_, the Li_2.9_Fe_0.9_Zr_0.1_Cl_6_ cell demonstrated better rate performance (Figure 4b, c). The discharge capacities are 70.4, 62.7, 59.5, 55.6, 46.7, and 36.1 mAh·g^–1^ at rates 0.1, 0.2, 0.5, 1, 2, and 3 C, respectively. The Li_2.9_Fe_0.9_Zr_0.1_Cl_6_ cells also show better cycling stability than the Li_3_FeCl_6_ cells as shown in the comparison of cycling performance under 0.5 C at 25 °C in Figures 3d and 4d. After 2000 cycles, the Li_2.9_Fe_0.9_Zr_0.1_Cl_6_ cell maintained a capacity retention of 80.4%.

**Figure 5:**
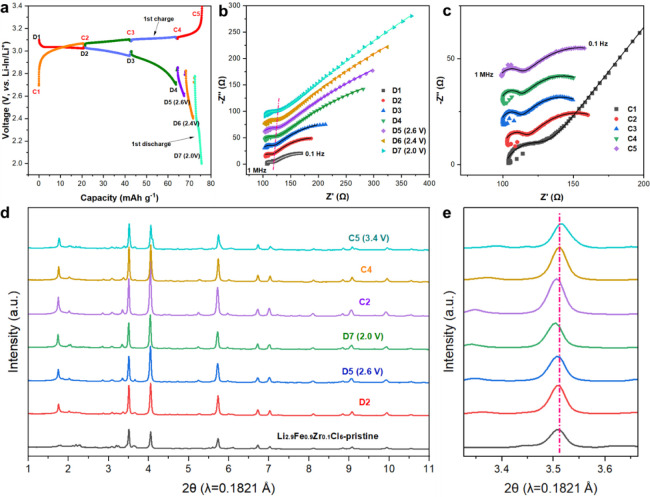
Impedance and XRD evolution of Li–In | Li_3_YCl_6_ | Li_2.9_Fe_0.9_Zr_0.1_Cl_6_ cells upon cycling. (a) First discharge and charge curves of Li–In | Li_3_YCl_6_ | Li_2.9_Fe_0.9_Zr_0.1_Cl_6_ cell at 25 °C. The cell was discharged/charged to different states at 0.1 C rate and then rested for 5 min. D1–D7 correspond to different states of discharge. D2, D3, and D4 are correlated with 25%, 50%, and 75% states of discharge, respectively. C1–C5 are corresponding to different states of charge. C2, C3, and C4 are correlated with 25%, 50%, and 75% states of charge, respectively. (b, c) Corresponding impedance plots recorded after 5 min rest during the discharge and charge process, respectively. (d) Ex situ XRD patterns of the Li_2.9_Fe_0.9_Zr_0.1_Cl_6_ positive electrode from the Li–In | Li_3_YCl_6_ | Li_2.9_Fe_0.9_Zr_0.1_Cl_6_ cell at different discharge/charge states. (e) Zoom-in on the XRD patterns corresponding to (d) to investigate the peak shift.

To further explore the evolution of ionic conductivity of Li_2.9_Fe_0.9_Zr_0.1_Cl_6_ as the function of Li concentration, EIS measurements were conducted using a Li–In | Li_3_YCl_6_ | Li_2.9_Fe_0.9_Zr_0.1_Cl_6_ cell and the results are shown in Figure 5a–c. The cell was discharged and then charged at a rate of 0.1 C rate. At each desired state of charge/discharge (SOC/SOD), EIS was collected after 5 min of rest. The spectra are fitted with equivalent circuits of R(RQ)(RQ) (Figure S9), where the resistance at 1 MHz is associated with the impedance of Li_3_YCl_6_ solid electrolyte. The resistance of the first semicircle in the spectra is attributed to interfacial resistance among Li_2.9_Fe_0.9_Zr_0.1_Cl_6_ particles. The second semicircle corresponds to charge transfer resistance. As the discharge process proceeds, the interfacial resistance only slightly increases from 13.8 to 25.5 Ω (Figure 5b), suggesting a stable interface of Li_2.9_Fe_0.9_Zr_0.1_Cl_6_ during the discharge process. The charge transfer resistance, however, increases significantly during the discharge process and gradually decreases as the charging goes on, indicating the reversibility of charge transfer resistance. EIS plots of different SODs of the second discharge process exhibit a similar trend to those of the first discharge process (Figure S10), further demonstrating the excellent stability of interface among Li_2.9_Fe_0.9_Zr_0.1_Cl_6_ particles.

Ex situ XRD patterns of Li_2.9_Fe_0.9_Zr_0.1_Cl_6_ at different SOCs and SODs were measured as well (Figure 5d). The XRD patterns of all ex situ Li_2.9_Fe_0.9_Zr_0.1_Cl_6_ samples can be indexed with the *C*2/*m* structure and no new phase is seen (Figure S11). While peak shifting toward lower 2-theta angles during discharging and shifting toward higher 2-theta during charging can be observed in Figure 5e, indicating a typical solid solution type phase evolution with the unit cell expansion in discharging and shrinkage in charging, corresponding to the Li insertion into and extraction from the lattice, respectively. The refined lattice parameters (*a* and *c*) and unit cell volume of Li_2.9_Fe_0.9_Zr_0.1_Cl_6_ gradually increase and then decrease upon Li^+^ insertion and extraction, as shown in Figure S12. The unit cell volume change of Li_2.9_Fe_0.9_Zr_0.1_Cl_6_ before and after Li^+^ insertion is merely 0.8%, which benefits from the complete solid-solution transition.

**Figure 6:**
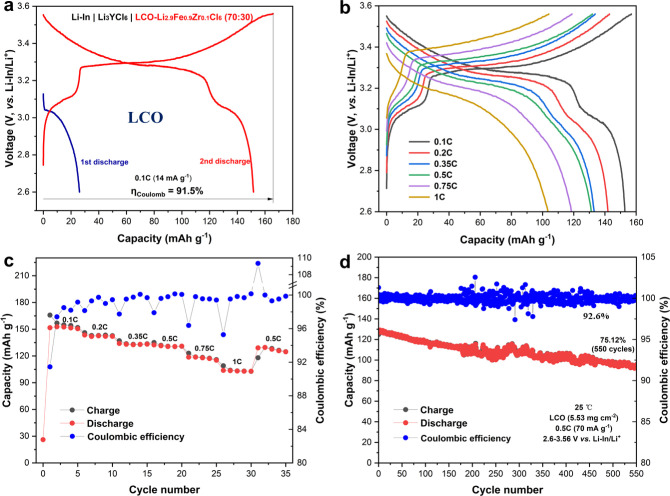
Electrochemical performance of Li–In | Li_3_YCl_6_ | LCO-Li_2.9_Fe_0.9_Zr_0.1_Cl_6_ (70:30) all-solid-state cells. All cells were tested within 2.6–3.56 V vs Li–In/Li^+^ at 25 °C. (a) Voltage profiles of the first discharge and following charge and discharge under 0.1 C (the specific capacity is calculated based on the loading of LCO and the practical capacity of LCO is ∼140 mAh·g^–1^ in this voltage range). (b, c) Rate capability at 0.1, 0.2, 0.35, 0.5, 0.75, and 1 C. (d) Long-term cycling performance under 0.5 C and 25 °C.

Li_2.9_Fe_0.9_Zr_0.1_Cl_6_ was then evaluated as a catholyte in the composite cathodes of ASSBs. Figure 6a shows the initial charge/discharge curves of the assembled ASSBs with a composite cathode of LCO and Li_2.9_Fe_0.9_Zr_0.1_Cl_6_ in a weight ratio of 70:30, a Li_3_YCl_6_ separator, and a Li–In anode cycled between 2.6 and 3.56 V versus Li–In/Li^+^ at a 0.1 C rate (1 C = 140 mA·g^–1^). The capacity is calculated based on the LCO loading amount in the cathode to facilitate the comparison with related reports. As shown in Figure 6a, Li_2.9_Fe_0.9_Zr_0.1_Cl_6_ contributes 26.1 mAh·g^–1^ capacity during the first discharge, and this portion of capacity remains stable during the following cycles. The good stability is also confirmed by EIS results (Figure S13). In the first charging curve, two voltage plateaus are seen. The one at ∼3.1 V corresponds to the Fe^2+^/Fe^3+^ redox in Li_2.9_Fe_0.9_Zr_0.1_Cl_6_, while the other one at 3.3 V corresponds to the Co^3+^/Co^4+^ redox in LCO. The total apparent specific capacity, calculated based on the mass of LCO, is 152.8 mAh·g^–1^ in the second discharge, much higher than those reported in ASSBs with LCO cathodes^8,16^ (Table S7), benefiting from the additional capacity contributed by the catholyte. The corresponding energy density is 399.6 Wh·kg^–1^ (based on the total mass of the composite cathode). Also benefiting from the good ionic conductivity and high weight percentage of the catholyte, excellent rate capability of the cells is achieved, as shown in Figure 6b and 6c. The discharge capacities are 152.8, 141.9, 133.1, 131.4, 118.7, and 103.5 mAh·g^–1^ at rates of 0.1, 0.2, 0.35, 0.5, 0.75, and 1 C, respectively. The outstanding cycling stability is shown in Figure 6d, where 75.12% capacity retention is achieved after 550 cycles under 0.5 C and 25 °C, offering an average Coulombic efficiency of 99.93%.

## 3 Conclusions

In summary, we report the use of Li_2.9_Fe_0.9_Zr_0.1_Cl_6_ as a catholyte to offer a higher energy density for all-solid-state batteries. Li_2.9_Fe_0.9_Zr_0.1_Cl_6_ with the *C*2/*m* space group exhibits good ionic/electronic conductivities (σ_Li+_: 0.25 mS·cm^–1^ and σ_e_: 2.16 × 10^–7^ S·cm^–1^ at 25 °C) and high specific capacity (71.4 mAh·g^–1^). The Li–In | Li_3_YCl_6_ | Li_2.9_Fe_0.9_Zr_0.1_Cl_6_ cell without additional SEs maintains a capacity retention of 80.4% over 2000 cycles, demonstrating the outstanding stability of Li_2.9_Fe_0.9_Zr_0.1_Cl_6_ upon cycling. ASSBs using the LCO cathode and Li_2.9_Fe_0.9_Zr_0.1_Cl_6_ catholyte demonstrate remarkable discharge capacity, as high as 152.8 mAh·g^–1^ at 0.1 C, excellent rate capability, and cycling performance. These findings shed light on a new strategy to increase energy density and reduce the costs for all-solid-state batteries. In addition, this work emphasizes the distinct requirements of SEs as catholytes and broadens the chemical space of the solid-electrolyte design, enabling the use of sustainable redox-active elements in catholytes.

## 4 Experimental Section

### Materials Synthesis

LiFeCl_4_, Li_2_FeCl_5_, Li_3_FeCl_6_, Li_4_FeCl_7_, and Li_3-*x*_Fe_1–*x*_Zr_*x*_Cl_6_ (*x* = 0.1, 0.2, and 0.3) were synthesized via solid-state reaction. Stoichiometric mixtures of anhydrous LiCl (>99%, Sigma-Aldrich), anhydrous FeCl_3_ (99.9%, Spectrum), and ZrCl_4_ (99%, Sigma-Aldrich) were weighted in an Ar-filled glovebox and ball-milled using zirconia jars (50 mL) in a planetary ball mill (PM 200, Retsch) at 300 rpm for 5h.

### Materials Characterization

X-ray diffraction patterns were collected at beamline 28-ID-2 (λ = 0.1821 Å) at the National Synchrotron Light Source II (NSLS II) at Brookhaven National Laboratory. The powders were sealed into a Kapton tube in an Ar-filled glovebox to avoid air exposure. Rietveld refinements against the XRD data were conducted using GSAS II.^42^ The Li–In | Li_3_YCl_6_ | Li_2.9_Fe_0.9_Zr_0.1_Cl_6_ cells utilized for the ex situ XRD measurements were assembled employing the same approach and parameters as those used for electrochemical performance tests. After the state of discharge/charge as shown in the manuscript was reached, the Li_2.9_Fe_0.9_Zr_0.1_Cl_6_ powders were collected and sealed into Kapton tube in an Ar-filled glovebox, and then the XRD measurement was performed at the National Synchrotron Light Source II (NSLS II) at Brookhaven National Laboratory.

### Conductivity Measurements

The electrochemical impedance spectroscopy (EIS) data were collected on Ti | SE | Ti symmetric cells using an electrochemical impedance analyzer (VMP3, BioLogic). The cold-pressed Li_3_FeCl_6_ or Li_2.9_Fe_0.9_Zr_0.1_Cl_6_ pellets were sandwiched by two pieces of Ti foils serving as current collectors. The EIS data were collected in the frequency range of 1 MHz to 0.1 Hz with an AC amplitude of 50 mV under an external pressure of 46.5 MPa. The electronic conductivities were measured through a direct current (DC) polarization measurement using an electrochemical workstation (VMP3, BioLogic) with an applied voltage of 0.1 V.

### Electrochemical Characterizations

The Li–In | Li_3_YCl_6_ | Li_3_FeCl_6_ and Li–In | Li_3_YCl_6_ | Li_2.9_Fe_0.9_Zr_0.1_Cl_6_ cells were assembled as following protocols. The cathode composite was made by mixing Li_3_FeCl_6_ or Li_2.9_Fe_0.9_Zr_0.1_Cl_6_ and carbon nanofiber in a mortar with a weight ratio of 95:5. Then the cathode composite was mixed using a vortex mixer (VM-3000, VWR) for 10 min.

In the assembly process, 130 mg of Li_3_YCl_6_ powder was pressed in a PTFE sleeve with 0.5 in. inner diameter under 243.0 MPa. Subsequently, 10 mg of cathode composite powder was homogeneously spread and pressed on the SE pellet using a pressure of 243.0 MPa. An In–Li alloy was affixed to the other side of the SE pellet as anode. Electrochemical performances were carried out in galvanostatic mode within 2.0–3.4 V vs Li–In/Li^+^ at 25 °C. Additionally, cyclic voltammetry (CV) measurement was conducted on the Li–In | Li_3_YCl_6_ | Li_3_FeCl_6_ cell. The data were obtained using an electrochemical impedance analyzer (VMP3, BioLogic) at a scan rate of 0.1 mV/s, ranging from 2.0 to 3.9 V vs Li–In/Li^+^.

For the assembly of Li–In | Li_3_YCl_6_ | LCO-Li_2.9_Fe_0.9_Zr_0.1_Cl_6_ cell, the protocol remains the same except for the composition of cathode composite and voltage window. The cathode composite comprised LCO, Li_2.9_Fe_0.9_Zr_0.1_Cl_6_, and carbon nanofiber in a weight ratio of 70:30:3. The cell was discharged/charged between 2.6 and 3.56 V vs Li–In/Li^+^.

## Supporting Information Available

BVSE map calculated by SoftBV; synchrotron XRD patterns; EIS spectra and transient current behavior of LiFeCl_4_, Li_2_FeCl_5_, Li_4_FeCl_7_, and Li_3-*x*_Fe_1–*x*_Zr_*x*_Cl_6_ (*x* = 0.1, 0.2, and 0.3); fitted diffraction patterns and corresponding Rietveld refinement results of Li_3_FeCl_6_, Li_2.9_Fe_0.9_Zr_0.1_Cl_6_, and discharged Li_2.9_Fe_0.9_Zr_0.1_Cl_6_; CV curve of Li_3_FeCl_6_ performed on the Li–In | Li_3_YCl_6_ | Li_3_FeCl_6_ cell; impedance plots of Li–In | Li_3_YCl_6_ | LCO-Li_2.9_Fe_0.9_Zr_0.1_Cl_6_ cell at different charge/discharge states; summary of cathode electrode components and electrochemical performances of ASSBs (PDF)

## References

[ref1] Famprikis T.; Canepa P.; Dawson J. A.; Islam M. S.; Masquelier C. Fundamentals of Inorganic Solid-State Electrolytes for Batteries. Nat. Mater. 2019, 18 (12), 1278–1291. 10.1038/s41563-019-0431-3. 31427742

[ref2] Zhou L.; Zuo T. T.; Kwok C. Y.; Kim S. Y.; Assoud A.; Zhang Q.; Janek J.; Nazar L. F. High Areal Capacity, Long Cycle Life 4 V Ceramic All-Solid-State Li-Ion Batteries Enabled by Chloride Solid Electrolytes. Nat. Energy 2022, 7 (1), 83–93. 10.1038/s41560-021-00952-0.

[ref3] Kato Y.; Hori S.; Saito T.; Suzuki K.; Hirayama M.; Mitsui A.; Yonemura M.; Iba H.; Kanno R. High-Power All-Solid-State Batteries Using Sulfide Superionic Conductors. Nat. Energy 2016, 1 (4), 16030 10.1038/nenergy.2016.30.

[ref4] Ma T.; Wang Z.; Wu D.; Lu P.; Zhu X.; Yang M.; Peng J.; Chen L.; Li H.; Wu F. High-Areal-Capacity and Long-Cycle-Life All-Solid-State Battery Enabled by Freeze Drying Technology. Energy Environ. Sci. 2023, 16 (5), 2142–2152. 10.1039/D3EE00420A.

[ref5] Janek J.; Zeier W. G. Challenges in Speeding up Solid-State Battery Development. Nat. Energy 2023, 8 (3), 230–240. 10.1038/s41560-023-01208-9.

[ref6] Asano T.; Sakai A.; Ouchi S.; Sakaida M.; Miyazaki A.; Hasegawa S. Solid Halide Electrolytes with High Lithium-Ion Conductivity for Application in 4 V Class Bulk-Type All-Solid-State Batteries. Adv. Mater. 2018, 30 (44), 1803075 10.1002/adma.201803075. 30216562

[ref7] Kwak H.; Han D.; Lyoo J.; Park J.; Jung S. H.; Han Y.; Kwon G.; Kim H.; Hong S.-T.; Nam K.-W.; Jung Y.-S. New Cost-Effective Halide Solid Electrolytes for All-Solid-State Batteries: Mechanochemically Prepared Fe^3+^-Substituted Li_2_ZrCl_6_. Adv. Energy Mater. 2021, 11 (12), 2003190 10.1002/aenm.202003190.

[ref8] Li X.; Liang J.; Luo J.; Banis M. N.; Wang C.; Li W.; Deng S.; Yu C.; Zhao F.; Hu Y.; Sham T. K.; Zhang L.; Zhao S.; Lu S.; Huang H.; Li R.; Adair K. R.; Sun X. Air-Stable Li_3_InCl_6_ Electrolyte with High Voltage Compatibility for All-Solid-State Batteries. Energy Environ. Sci. 2019, 12 (9), 2665–2671. 10.1039/C9EE02311A.

[ref9] Liang J.; Li X.; Kim J. T.; Hao X.; Duan H.; Li R.; Sun X. Halide Layer Cathodes for Compatible and Fast-Charged Halides-Based All-Solid-State Li Metal Batteries. Angew. Chem., Int. Ed. 2023, 62 (13), e202217081 10.1002/anie.202217081. 36697365

[ref10] Wang C.; Liang J.; Zhao Y.; Zheng M.; Li X.; Sun X. All-Solid-State Lithium Batteries Enabled by Sulfide Electrolytes: From Fundamental Research to Practical Engineering Design. Energy Environ. Sci. 2021, 14 (5), 2577–2619. 10.1039/D1EE00551K.

[ref11] Wang C.; Liang J.; Luo J.; Liu J.; Li X.; Zhao F.; Li R.; Huang H.; Zhao S.; Zhang L.; Wang J.; Sun X. A Universal Wet-Chemistry Synthesis of Solid-State Halide Electrolytes for All-Solid-State Lithium-Metal Batteries. Sci. Adv. 2021, 7 (37), eabh1896 10.1126/sciadv.abh1896. 34516879 PMC8442915

[ref12] Jin Z.; Kong X.; Huang H.; Jiang Y.; Xiang W.; Xu Y.; Zhang L.; Peng R.; Wang C. Garnet-Type Solid-State Mixed Ionic and Electronic Conductor. Energy Storage Mater. 2023, 59, 102788 10.1016/j.ensm.2023.102788.

[ref13] Xiao Y.; Wang Y.; Bo S. H.; Kim J. C.; Miara L. J.; Ceder G. Understanding Interface Stability in Solid-State Batteries. Nat. Rev. Mater. 2020, 5 (2), 105–126. 10.1038/s41578-019-0157-5.

[ref14] Li X.; Liang J.; Yang X.; Adair K. R.; Wang C.; Zhao F.; Sun X. Progress and Perspectives on Halide Lithium Conductors for All-Solid-State Lithium Batteries. Energy Environ. Sci. 2020, 13 (5), 1429–1461. 10.1039/C9EE03828K.

[ref15] Liu Z.; Zinkevich T.; Indris S.; He X.; Liu J.; Xu W.; Bai J.; Xiong S.; Mo Y.; Chen H. Li_15_P_4_S_16_Cl_3_, a Lithium Chlorothiophosphate as a Solid-State Ionic Conductor. Inorg. Chem. 2020, 59 (1), 226–234. 10.1021/acs.inorgchem.9b01751. 31829567

[ref16] Liu Z.; Ma S.; Liu J.; Xiong S.; Ma Y.; Chen H. High Ionic Conductivity Achieved in Li_3_Y(Br_3_Cl_3_) Mixed Halide Solid Electrolyte via Promoted Diffusion Pathways and Enhanced Grain Boundary. ACS Energy Lett. 2021, 6 (1), 298–304. 10.1021/acsenergylett.0c01690.

[ref17] Tanaka Y.; Ueno K.; Mizuno K.; Takeuchi K.; Asano T.; Sakai A. New Oxyhalide Solid Electrolytes with High Lithium Ionic Conductivity > 10 mS Cm^–1^ for All-Solid-State Batteries. Angew. Chem., Int. Ed. 2023, 62 (13), e202217581 10.1002/anie.202217581. 36747340

[ref18] Li F.; Cheng X.; Lu G.; Yin Y. C.; Wu Y. C.; Pan R.; Luo J. D.; Huang F.; Feng L. Z.; Lu L. L.; et al. Amorphous Chloride Solid Electrolytes with High Li-Ion Conductivity for Stable Cycling of All-Solid-State High-Nickel Cathodes. J. Am. Chem. Soc. 2023, 145 (50), 27774–27787. 10.1021/jacs.3c10602. 38079498

[ref19] Yin Y. C.; Yang J. T.; Luo J. D.; Lu G. X.; Huang Z.; Wang J. P.; Li P.; Li F.; Wu Y. C.; Tian T.; et al. A LaCl3-Based Lithium Superionic Conductor Compatible with Lithium Metal. Nature 2023, 616 (7955), 77–83. 10.1038/s41586-023-05899-8. 37020008

[ref20] Han F.; Westover A. S.; Yue J.; Fan X.; Wang F.; Chi M.; Leonard D. N.; Dudney N. J.; Wang H.; Wang C. High Electronic Conductivity as the Origin of Lithium Dendrite Formation within Solid Electrolytes. Nat. Energy 2019, 4 (3), 187–196. 10.1038/s41560-018-0312-z.

[ref21] Song Y.; Yang L.; Zhao W.; Wang Z.; Zhao Y.; Wang Z.; Zhao Q.; Liu H.; Pan F. Revealing the Short-Circuiting Mechanism of Garnet-Based Solid-State Electrolyte. Adv. Energy Mater. 2019, 9 (21), 1900671 10.1002/aenm.201900671.

[ref22] Al-Salih H.; Houache M. S. E.; Baranova E. A.; Abu-Lebdeh Y. Composite Cathodes for Solid-State Lithium Batteries: “Catholytes” the Underrated Giants. Adv. Energy Sustainability Res. 2022, 3 (8), 2200032 10.1002/aesr.202200032.

[ref23] Papakyriakou M.; Lu M.; Liu Y.; Liu Z.; Chen H.; McDowell M. T.; Xia S. Mechanical Behavior of Inorganic Lithium-Conducting Solid Electrolytes. J. Power Sources 2021, 516, 230672 10.1016/j.jpowsour.2021.230672.

[ref24] Wang K.; Gu Z.; Xi Z.; Hu L.; Ma C. Li_3_TiCl_6_ as Ionic Conductive and Compressible Positive Electrode Active Material for All-Solid-State Lithium-Based Batteries. Nat. Commun. 2023, 14 (1), 1396 10.1038/s41467-023-37122-7. 36914653 PMC10011600

[ref25] Hennequart B.; Platonova M.; Chometon R.; Marchandier T.; Benedetto A.; Quemin E.; Dugas R.; Lethien C.; Tarascon J. M. Atmospheric-Pressure Operation of All-Solid-State Batteries Enabled by Halide Solid Electrolyte. ACS Energy Lett. 2024, 9 (2), 454–460. 10.1021/acsenergylett.3c02513.

[ref26] Palvadeau P.; Venien J. P.; Spiesser M.; Rouxel J. Characterization of LiFeCl_4_ and AgFeCl_4_ Ionic Conductors. Solid State Ion. 1982, 6 (3), 231–236. 10.1016/0167-2738(82)90044-3.

[ref27] Wang S.; Bai Q.; Nolan A. M.; Liu Y.; Gong S.; Sun Q.; Mo Y. Lithium Chlorides and Bromides as Promising Solid-State Chemistries for Fast Ion Conductors with Good Electrochemical Stability. Angew. Chem., Int. Ed. 2019, 58 (24), 8039–8043. 10.1002/anie.201901938. 30977261

[ref28] Liang J.; Li X.; Wang S.; Adair K. R.; Li W.; Zhao Y.; Wang C.; Hu Y.; Zhang L.; Zhao S.; et al. Site-Occupation-Tuned Superionic Li_x_ScCl_3+x_ Halide Solid Electrolytes for All-Solid-State Batteries. J. Am. Chem. Soc. 2020, 142 (15), 7012–7022. 10.1021/jacs.0c00134. 32212650

[ref29] Kwak H.; Han D.; Son J. P.; Kim J. S.; Park J.; Nam K. W.; Kim H.; Jung Y. S. Li^+^ Conduction in Aliovalent-Substituted Monoclinic Li_2_ZrCl_6_ for All-Solid-State Batteries: Li_2+x_Zr_1-x_M_x_Cl_6_ (M = In, Sc). Chem. Eng. J. 2022, 437, 135413 10.1016/j.cej.2022.135413.

[ref30] Chen H.; Adams S. Bond Softness Sensitive Bond-valence Parameters for Crystal Structure Plausibility Tests. Iucr J. 2017, 4, 614–625. 10.1107/S2052252517010211. PMC5619853 28989717

[ref31] Chen H.; Wong L. L.; Adams S. SoftBV–a Software Tool for Screening the Materials Genome of Inorganic Fast Ion Conductors. Acta Crystallogr. 2019, B75, 18–33. 10.1107/S2052520618015718. 32830774

[ref32] Momma K.; Izumi F. VESTA 3 for Three-dimensional Visualization of Crystal, Volumetric and Morphology Data. J. Appl. Crystallogr. 2011, 44, 1272–1276. 10.1107/S0021889811038970.

[ref33] Flores-González N.; López M.; Minafra N.; Bohnenberger J.; Viñes F.; Rudić S.; Krossing I.; Zeier W. G.; Illas F.; Gregory D. H. Understanding the Effect of Lattice Polarisability on the Electrochemical Properties of Lithium Tetrahaloaluminates, LiAlX_4_ (X = Cl, Br, I). J. Mater. Chem. A 2022, 10 (25), 13467 10.1039/D2TA02821B.

[ref34] Hu L.; Wang J.; Wang K.; Gu Z.; Xi Z.; Li H.; Chen F.; Wang Y.; Li Z.; Ma C. A Cost-Effective, Ionically Conductive and Compressible Oxychloride Solid-State Electrolyte for Stable All-Solid-State Lithium-Based Batteries. Nat. Commun. 2023, 14 (1), 3807 10.1038/s41467-023-39522-1. 37369677 PMC10300059

[ref35] Park K. H.; Kaup K.; Assoud A.; Zhang Q.; Wu X.; Nazar L. F. High-Voltage Superionic Halide Solid Electrolytes for All-Solid-State Li-Ion Batteries. ACS Energy Lett. 2020, 5 (2), 533–539. 10.1021/acsenergylett.9b02599.

[ref36] Fu J.; Yang S.; Hou J.; Azhari L.; Yao Z.; Ma X.; Liu Y.; Vanaphuti P.; Meng Z.; Yang Z.; et al. Modeling Assisted Synthesis of Zr-Doped Li_3-x_In_1-x_ZrxCl_6_ with Ultrahigh Ionic Conductivity for Lithium-Ion Batteries. J. Power Sources 2023, 556, 232465 10.1016/j.jpowsour.2022.232465.

[ref37] Helm B.; Schlem R.; Wankmiller B.; Banik A.; Gautam A.; Ruhl J.; Li C.; Hansen M. R.; Zeier W. G. Exploring Aliovalent Substitutions in the Lithium Halide Superionic Conductor Li_3–_*_x_* In_1–_*_x_*Zr*_x_*Cl _6_ (0 ≤ *x* ≤ 0.5). Chem. Mater. 2021, 33 (12), 4773–4782. 10.1021/acs.chemmater.1c01348.

[ref38] Xiong S.; Liu Z.; Yang L.; Ma Y.; Xu W.; Bai J.; Chen H. Anion and Cation Co-Doping of Na_4_SnS_4_ as Sodium Superionic Conductors. Mater. Today Phys. 2020, 15, 100281 10.1016/j.mtphys.2020.100281.

[ref39] Xiong S.; Liu Z.; Rong H.; Wang H.; McDaniel M.; Chen H. Na_3_SbSe_4–x_S_x_ as Sodium Superionic Conductors. Sci. Rep. 2018, 8 (1), 9146–9153. 10.1038/s41598-018-27301-8. 29904054 PMC6002371

[ref40] Han Y.; Jung S. H.; Kwak H.; Jun S.; Kwak H. H.; Lee J. H.; Hong S.-T.; Jung Y. S. Single- or Poly-Crystalline Ni-Rich Layered Cathode, Sulfide or Halide Solid Electrolyte: Which Will Be the Winners for All-Solid-State Batteries?. Adv. Energy Mater. 2021, 11 (21), 2100126 10.1002/aenm.202100126.

[ref41] Kim S. Y.; Cha H.; Kostecki R.; Chen G. Composite Cathode Design for High-Energy All-Solid-State Lithium Batteries with Long Cycle Life. ACS Energy Lett. 2023, 8 (1), 521–528. 10.1021/acsenergylett.2c02414.

[ref42] Toby B. H.; Von Dreele R. B. GSAS-II: The Genesis of a Modern Open-Source All Purpose Crystallography Software Package. J. Appl. Crystallogr. 2013, 46 (2), 544–549. 10.1107/S0021889813003531.

