# Supplementary material for: Li2.9Fe0.9Zr0.1Cl6 as Redox-Active Catholyte for Solid-State Li-Ion Batteries
Source: Chem Mater. 2024 Oct 7;36(20):10104–12. doi: 10.1021/acs.chemmater.4c01385 (PMC11500307; doi:10.1021/acs.chemmater.4c01385)
Supplement: Supplementary file 1 — cm4c01385_si_001.pdf [file cm4c01385_si_001.pdf]

Supplementary Information

$Li_{2.9}Fe_{0.9}Zr_{0.1}Cl_6$ as Redox-active Catholyte for Solid-state Li-ion Batteries

Guangxing Zhang[1], Zhantao Liu[1], Yifan Ma[1], Jakub Pepas[1,2], Jianming Bai[3], Hui Zhong[3], Yuanzhi Tang[4]*, Hailong Chen[1]*

1. *The Woodruff School of Mechanical Engineering, Georgia Institute of Technology, Atlanta, GA, 30332, United States*
2. *School of Materials Science and Engineering, Georgia Institute of Technology, Atlanta, GA, 30332, United States*
3. *National Synchrotron Light Source II, Brookhaven National Laboratory, Upton, NY, 11973, United States*
4. *School of Earth and Atmospheric Sciences, Georgia Institute of Technology, Atlanta, GA, 30332, United States*

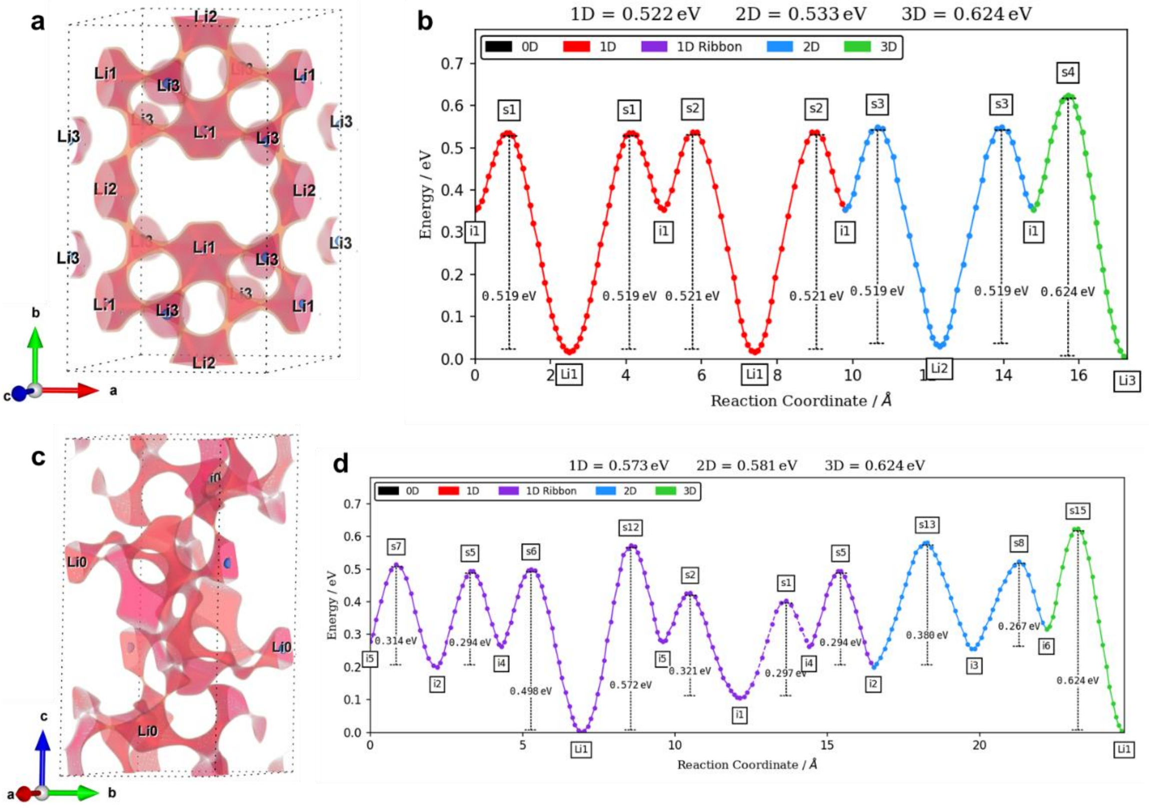

Figure S1. **(a)** BVSE map showing Li^+ migration pathways in a (001) projection and **(b)** BVSE model of migration barriers of *C*2/*m* Li_3FeCl_6 derived from Rietveld refinement result. **(c)** BVSE map showing Li^+ migration pathways in a (100) projection and **(d)** BVSE model of migration barriers of *P*2_1/*c* $LiFeCl_4$ derived from Rietveld refinement result. BVSE maps are visualized with VESTA.

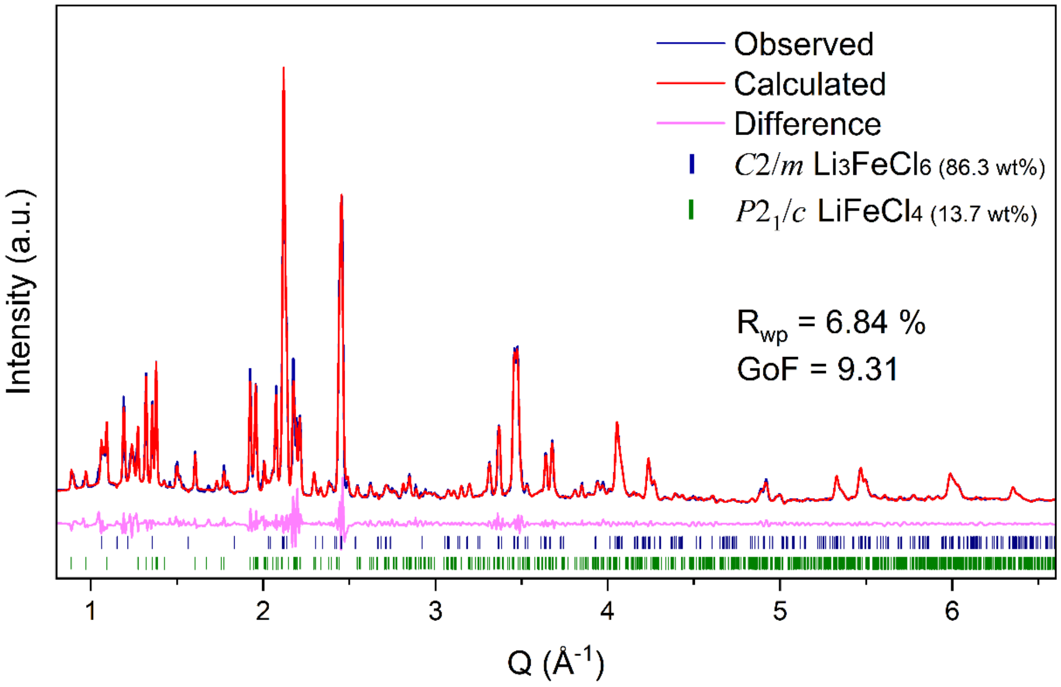

Figure S2. Synchrotron X-ray diffraction pattern of Li_3FeCl_6 and the corresponding Rietveld refinement.

Table S1. Rietveld refinement result of as-milled Li_3FeCl_6 using synchrotron XRD data.

Phase1: Li_3FeCl_6 (*C*2/*m*)					Phase weight ratio: 86.3%	
a = 6.244(1) Å		b = 10.9123(4) Å	c = 6.285(1) Å	β = 109.596(3)°	V = 403.525(34) $Å^3$	
Atom	Wyck.	x	y	z	Occ.	U_{iso} ($Å^2$)
Li1	4h	0	0.171(8)	0.5	0.391(5)	0.085(3)
Li2	2d	0.5	0	0.5	0.218(1)	0.075(8)
Li3	4g	0.5	0.825(1)	0	1	0.008(3)
Fe1	2a	0	0	0	1	0.134(2)
Cl1	4i	0.2392(2)	0.8388(1)	0.2366(2)	1	0.0187(9)
Cl2	8j	0.7589(3)	0	0.2327(4)	1	0.014(1)

Phase2: $LiFeCl_4$ (*P*2_1/c)					Phase weight ratio: 13.7%	
a = 7.1051(4) Å		b = 6.4228(3) Å	c = 12.9549(7) Å	β = 93.637(3)°	V = 590.001(43) $Å^3$	
Atom	Wyck.	x	y	z	Occ.	U_{iso} ($Å^2$)
Li1	4e	0.146(8)	0.505(8)	0.872(4)	1	0.106(2)
Fe1	4e	0.2949(6)	0.6760(5)	0.1014(3)	1	0.033(2)
Cl1	4e	0.0674(7)	0.6824(8)	0.6910(3)	1	0.013(2)
Cl2	4e	0.1851(7)	0.1383(7)	0.5654(4)	1	0.016(2)
Cl3	4e	0.3096(6)	0.6789(7)	0.4467(4)	1	0.013(2)
Cl4	4e	0.4309(6)	0.1739(7)	0.3106(3)	1	0.015(2)

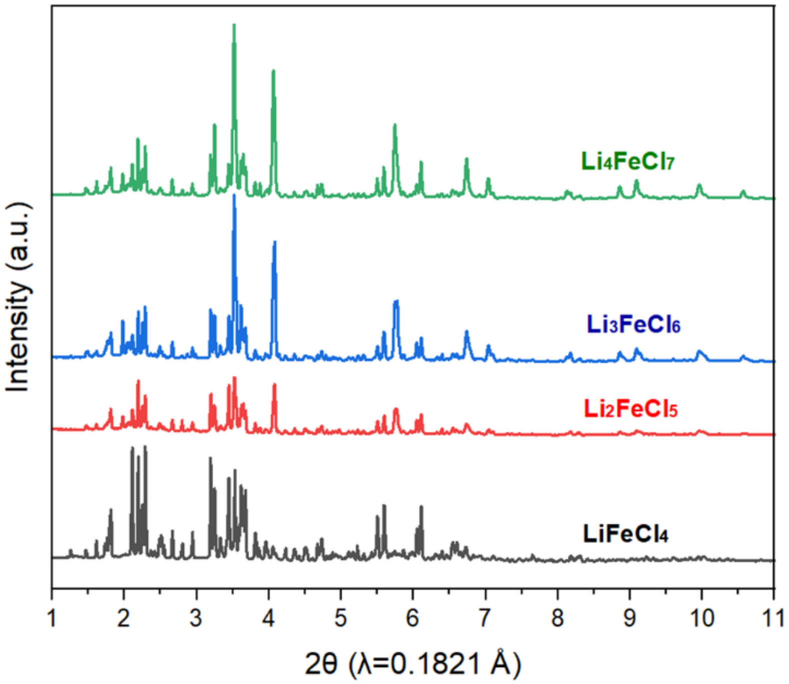

Figure S3. Synchrotron XRD patterns of $LiFeCl_4$, Li_2FeCl_5, Li_3FeCl_6 and Li_4FeCl_7.

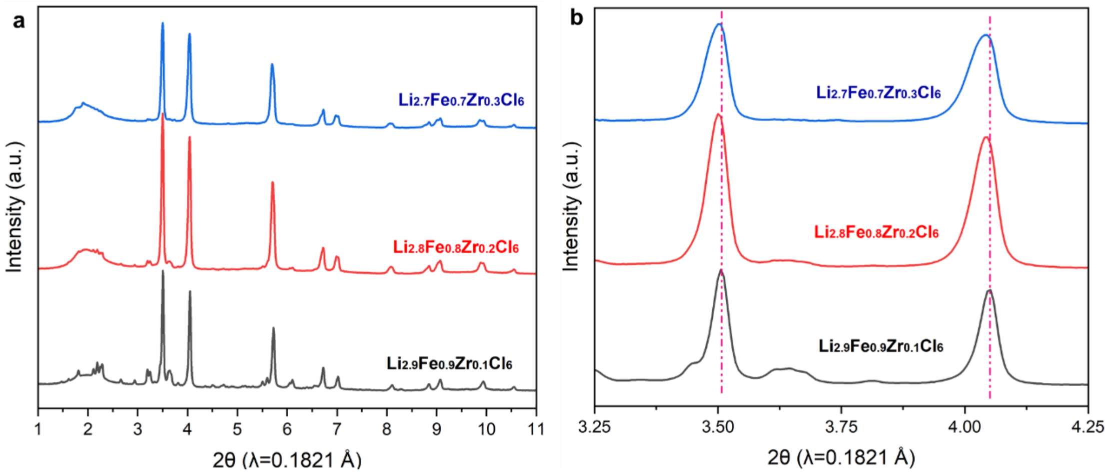

Figure S4. (a) Synchrotron XRD patterns of $Li_{3-x}Fe_{1-x}Zr_xCl_6$ (x = 0.1, 0.2 and 0.3). **(b)** Magnified XRD patterns from 3.25° to 4.25°, showing the peak shift among $Li_{3-x}Fe_{1-x}Zr_xCl_6$ samples.

Table S2. Rietveld refinement result of as-milled $Li_{2.9}Fe_{0.9}Zr_{0.1}Cl_6$ using synchrotron XRD data.

Phase1: Li_3FeCl_6 (*C*2/*m*)				Phase weight ratio: 97.6%		
a = 6.2962(4) Å	b = 10.9391(2) Å		c = 6.2863(5) Å	β = 109.442(1)°	V = 408.280(24) $Å^3$	
Atom	Wyck.	x	y	z	Occ.	U_{iso} ($Å^2$)
Li1	4h	0	0.130(2)	0.5	0.341(2)	0.009(6)
Li2	2d	0.5	0	0.5	0.267(4)	0.009(1)
Li3	4g	0.5	0.8318(9)	0	0.9926(3)	0.013(4)
Fe1	2a	0	0	0	0.912(3)	0.228(1)
Zr1	2a	0	0	0	0.088(3)	0.142(3)
Cl1	4i	0.2524(1)	0.8340(1)	0.2450(3)	1	0.0100(4)
Cl2	8j	0.7467(2)	0	0.2660(3)	1	0.0194(6)

Phase2: $LiFeCl_4$ ($P2_1$/c)				Phase weight ratio: 2.4%		
a = 7.0709(4) Å	b = 6.4380(3) Å		c = 12.8799(7) Å	β = 94.205(3)°	V = 584.739(48) $Å^3$	
Atom	Wyck.	x	y	z	Occ.	U_{iso} ($Å^2$)
Li1	4e	0.116(1)	0.483(1)	0.8636(8)	1	0.009(3)
Fe1	4e	0.2964(2)	0.6786(2)	0.1019(1)	1	0.0104(3)
Cl1	4e	0.0580(7)	0.674(1)	0.6886(4)	1	0.009(1)
Cl2	4e	0.1828(9)	0.140(1)	0.5625(4)	1	0.014(2)
Cl3	4e	0.3052(8)	0.671(1)	0.4443(4)	1	0.012(1)
Cl4	4e	0.4172(7)	0.166(1)	0.3128(5)	1	0.010(1)

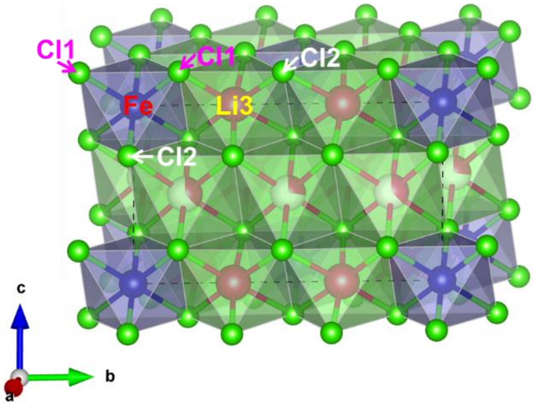

Figure S5. Crystal structures of Li_3FeCl_6 (*C*2/*m*) projected along the a-axis, respectively. Fe, Li, and Cl are represented by blue, pink, and green colored spheres, respectively.

Table S3. Rietveld bond length parameters for *C*2/*m* phase of Li_3FeCl_6 and $Li_{2.9}Fe_{0.9}Zr_{0.1}Cl_6$ sample using synchrotron XRD data.

**samples**	**Interatomic distance (Å)**			
	Fe−Cl1	Fe−Cl2	Li3−Cl1	Li3−Cl2
Li_3FeCl_6	2.459	2.425	2.474	2.616
$Li_{2.9}Fe_{0.9}Zr_{0.1}Cl_6$	2.563	2.668	2.549	2.631

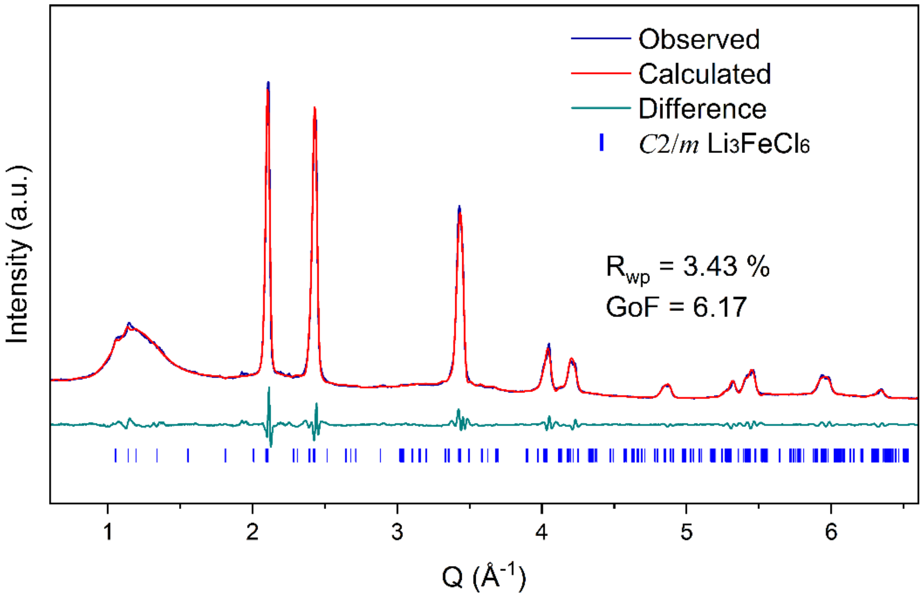

Figure S6. Synchrotron X-ray diffraction pattern of $Li_{2.7}Fe_{0.7}Zr_{0.3}Cl_6$ and the corresponding Rietveld refinement.

Table S4. Rietveld refinement result of as-milled $Li_{2.7}Fe_{0.7}Zr_{0.3}Cl_6$ using synchrotron XRD data.

Phase: Li_3FeCl_6 (*C*2/*m*)						
a = 6.360(3) Å	b = 11.060(2) Å	c = 6.321(3) Å	β = 109.782(8)°	V = 418.438(2) Å^3		
Atom	Wyck.	x	y	z	Occ.	U_{iso} (Å^2)
Li1	4h	0	0.124(4)	0.5	0.300(2)	0.102(9)
Li2	2d	0.5	0	0.5	0.372(4)	0.757(5)
Li3	4g	0.5	0.865(1)	0	0.857(1)	0.055(4)
Fe1	2a	0	0	0	0.770(5)	0.102(8)
Zr1	2a	0	0	0	0.230(5)	0.110(1)
Cl1	4i	0.248(1)	0.8358(6)	0.242(1)	1	0.018(6)
Cl2	8j	0.751(2)	0	0.264(2)	1	0.040(8)

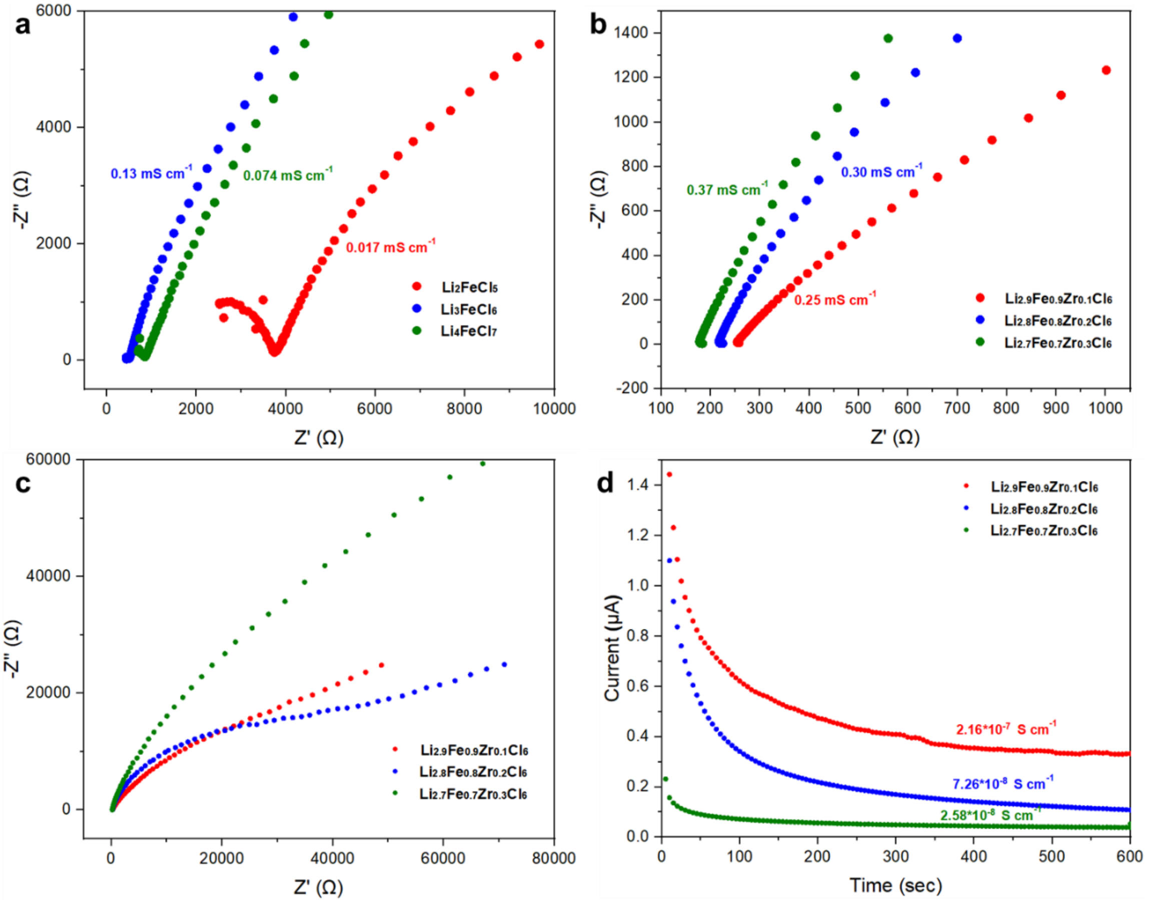

Figure S7. (a) Nyquist plots of the as-milled Li_2FeCl_5, Li_3FeCl_6 and Li_4FeCl_7 at 25 °C. **(b-c)** The enlarged and complete impedance plots of the as-milled $Li_{2.9}Fe_{0.9}Zr_{0.1}Cl_6$, $Li_{2.8}Fe_{0.8}Zr_{0.2}Cl_6$ and

$Li_{2.7}Fe_{0.7}Zr_{0.3}Cl_6$ at 25 °C. **(d)** The transient current behavior under an applied 0.1 V DC bias on $Li_{2.9}Fe_{0.9}Zr_{0.1}Cl_6$, $Li_{2.8}Fe_{0.8}Zr_{0.2}Cl_6$ and $Li_{2.7}Fe_{0.7}Zr_{0.3}Cl_6$.

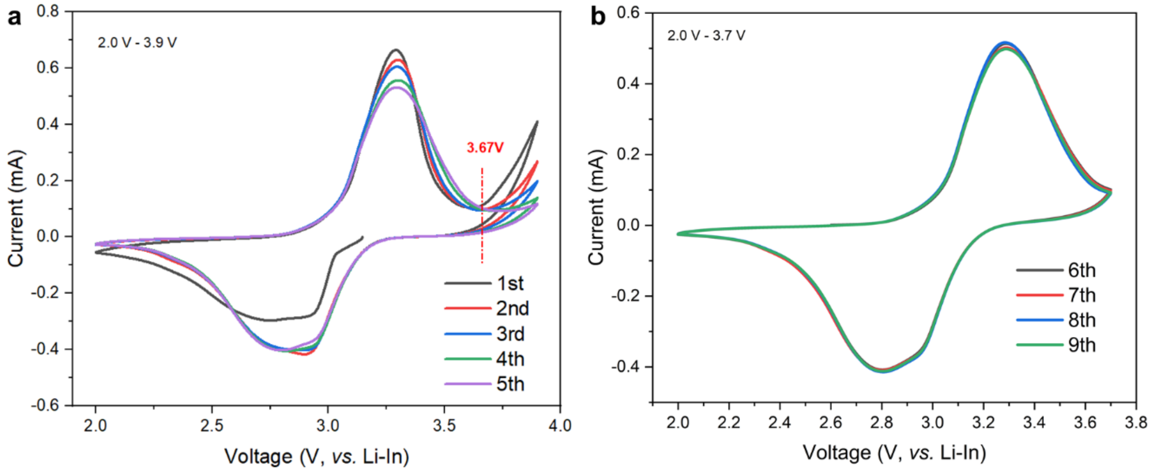

Figure S8. Cyclic voltammogram (CV) of Li_3FeCl_6 at a scan rate of 0.1 mV/s. The CV measurements were performed on the Li-In | Li_3YCl_6 | Li_3FeCl_6 cell. **(a)** CV spectra of the first five cycles performed within 2.0-3.9 V *vs.* Li-In/Li^+. **(b)** CV curves cycles 6 to 9 tested within 2.0-3.7 V *vs.* Li-In/Li^+.

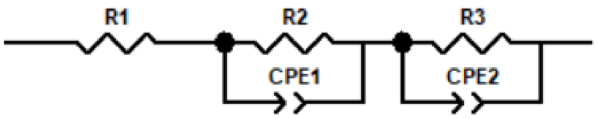

Figure S9. Equivalent circuit of R(RQ)(RQ) for impedance spectra in Figure 5b-c.

Table S5. Fitting results for impedance spectra in Figure 5b-c of Li-In | Li_3YCl_6 | $Li_{2.9}Fe_{0.9}Zr_{0.1}Cl_6$ cell during charging/discharging process.

		**Fitting results**		
		**R1 (Ω)**	**R2 (Ω)**	**R3 (Ω)**
**Discharging**	D1	102.9	13.8	110.1
	D2	99.8	18.7	152.2
	D3	100.3	18.6	234.2
	D4	99.1	26.2	674.4
	D5 (2.6 V)	100.6	24.1	1020.0
	D6 (2.4 V)	100.6	24.8	1699.0
	D7 (2.0 V)	100.9	25.5	2996.0
**Charging**	C1	100.9	25.5	2996.0
	C2	100.7	15.4	73.2
	C3	98.0	13.9	59.5
	C4	98.0	14.0	62.0

	C5	96.1	16.8	84.5

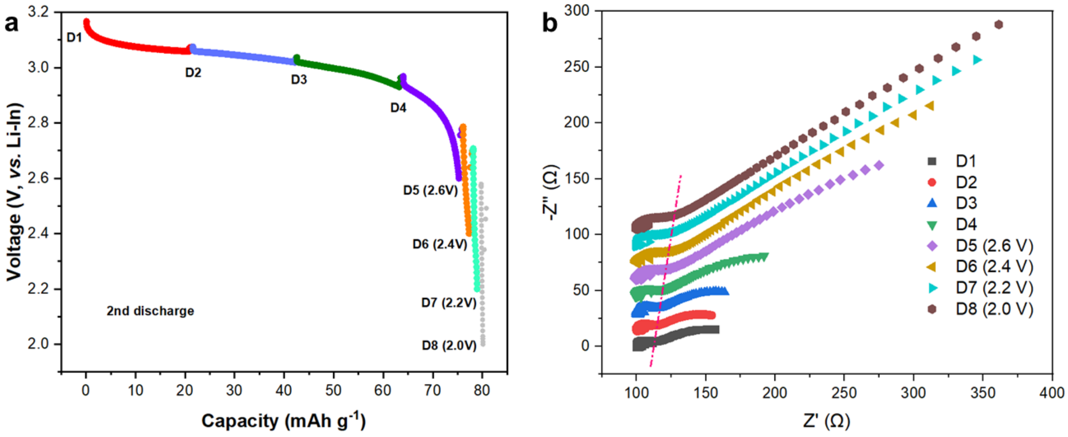

Figure S10. (a) Second discharge curve of Li-In | Li_3YCl_6 | $Li_{2.9}Fe_{0.9}Zr_{0.1}Cl_6$ cell under 0.1 C. **(b)** Impedance plots of the conductivity data during second discharge process.

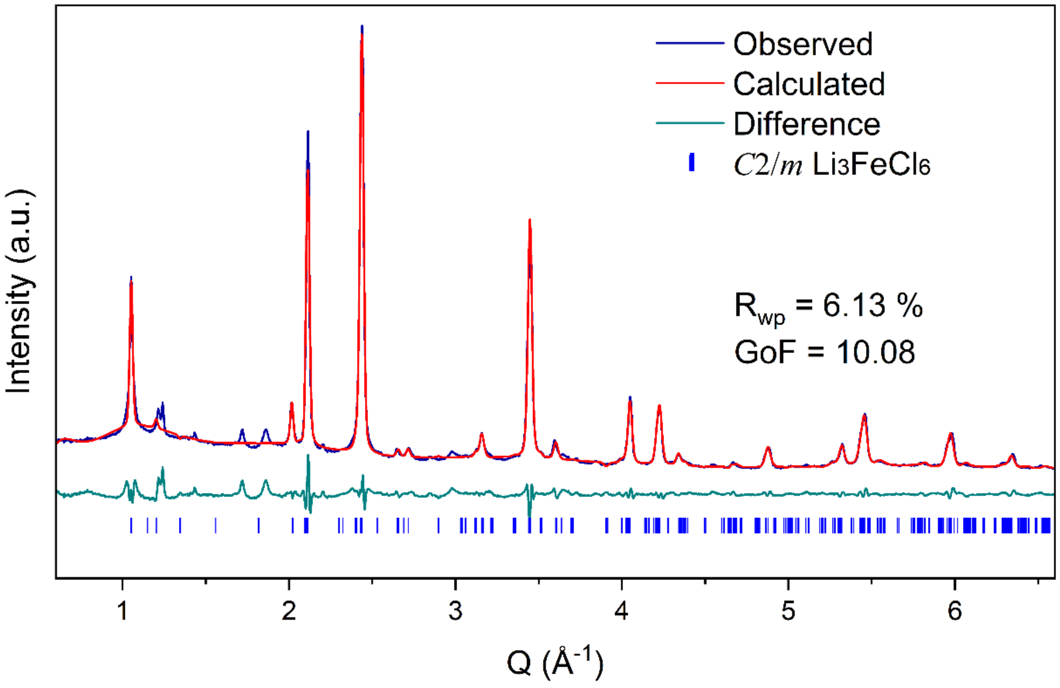

Figure S11. Synchrotron X-ray diffraction pattern of discharged $Li_{2.9}Fe_{0.9}Zr_{0.1}Cl_6$ to 2.0 V and the corresponding Rietveld refinement.

Table S6. Rietveld refinement result of the discharged $Li_{2.9}Fe_{0.9}Zr_{0.1}Cl_6$ to 2.0 V using synchrotron XRD data.

Phase: Li_3FeCl_6 (*C*2/*m*)						
a = 6.314(2) Å		b = 10.9259(7) Å	c = 6.335(2) Å	β = 109.636(6)°	V = 411.615(39) Å^3	
Atom	Wyck.	x	y	z	Occ.	U_{iso} (Å^2)
Li1	4h	0	0.183(7)	0.5	0.678(2)	0.009(2)
Li2	2d	0.5	0	0.5	0.644(5)	0.251(9)
Li3	4g	0.5	0.821(1)	0	0.708(6)	0.169(8)
Li4	2a	0	0	0	0.585(1)	0.46(0)
Fe1	2a	0	0	0	0.315(1)	0.393(0)
Fe2	4g	0.5	0.821(1)	0	0.292(6)	0.026(7)
Zr1	2a	0	0	0	0.1	0.025(1)
Cl1	4i	0.2447(5)	0.8369(8)	0.2417(5)	1	0.037(2)
Cl2	8j	0.7499(7)	0	0.2258(5)	1	0.004(2)

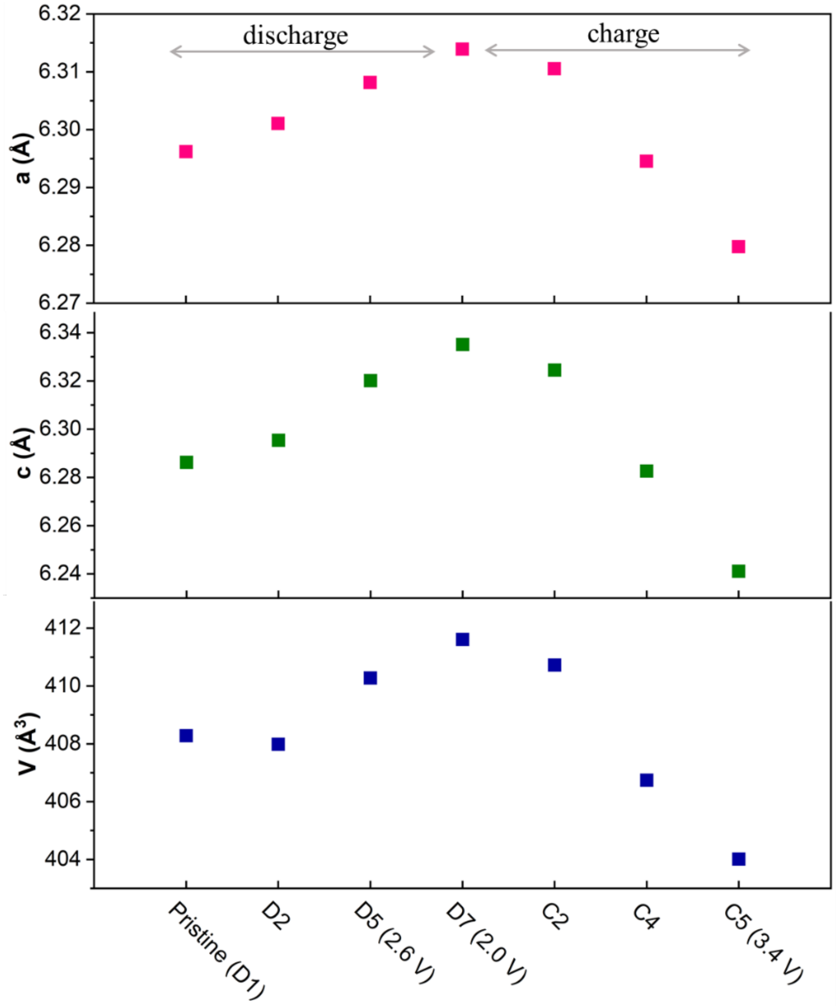

Figure S12. Variation of lattice constants and unit cell volume during cycling. The variation of lattice constants and unit-cell volume along with lithium insertion (upon discharge) and extraction (upon

charge) for $Li_{2.9}Fe_{0.9}Zr_{0.1}Cl_6$. These values were estimated from electrochemical ex situ XRD results as shown in **Fig. 5d**.

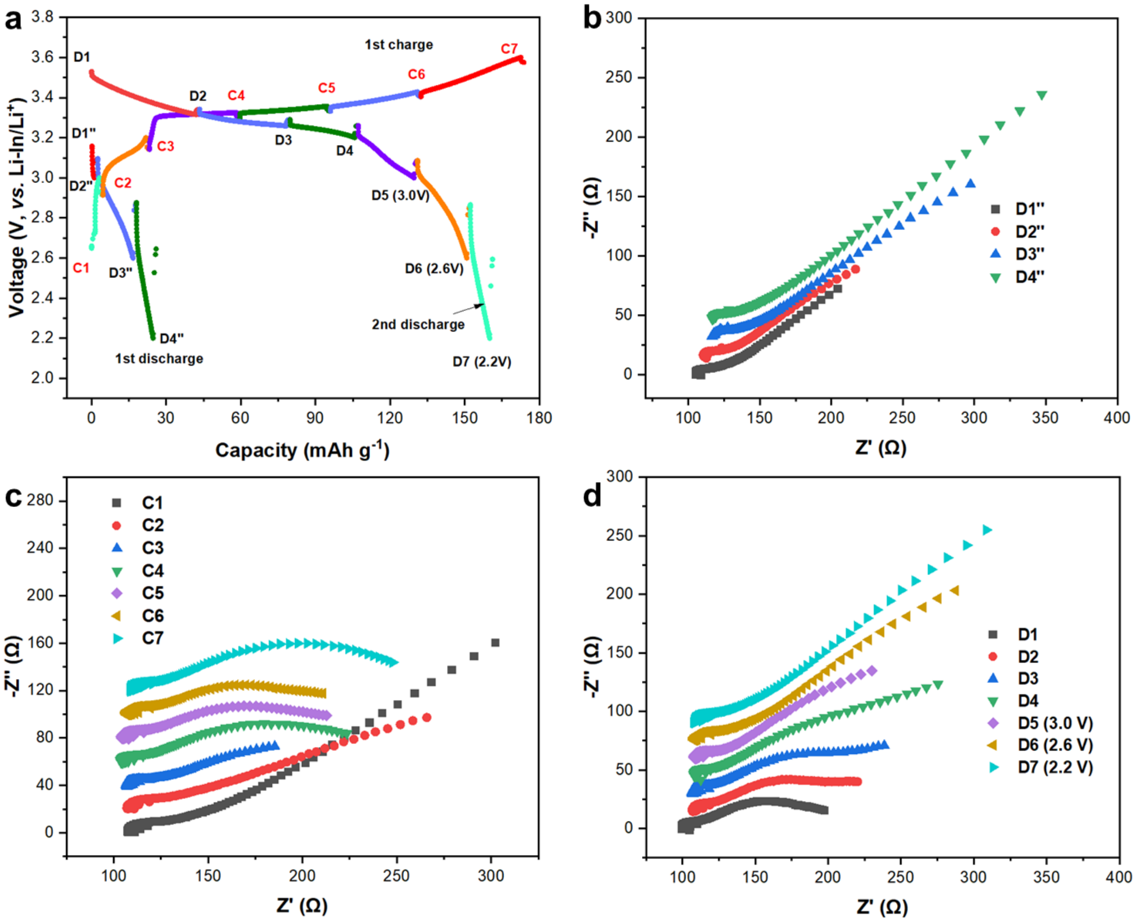

Figure S13. (a) Voltage profiles of the first discharge and following charge and discharge of Li-In | Li_3YCl_6 | LCO-$Li_{2.9}Fe_{0.9}Zr_{0.1}Cl_6$ cell under 0.1 C. **(b, c)** Impedance plots of the conductivity data during first discharge and charge process, respectively. **(d)** Nyquist plots of the cell during the second discharge process.

Table S7. Summary of cathode electrode components and electrochemical performances of ASSBs. The capacity is calculated based on the LCO mass loading in the cathode electrode to facilitate the fair comparison with related reports. The c-rate and working temperature for the studies below are 0.1 C and 25 °C, respectively. Carbon black has been added into the cathode electrode in some reports, resulting in the sum of the LCO and catholyte loading being less than 100% in certain cases.

References:

Composite cathode	Anode	Anodic interlayer	Solid electrolyte	Weight fraction of LCO [%]	Weight fraction of catholyte [%]	Voltage range [V vs Li/Li^+]	First discharge capacity [mAh g^{-1}]		Total discharge capacity normalized to the mass of cathode electrode [mAh g^{-1}]	Refs.
							capacity contributed by CAM	capacity from catholyte		
LCO/ $Li_{2.9}Fe_{0.9}Zr_{0.1}Cl_6$	Li-In	/	Li_3YCl_6	68	29	3.22−4.18	126.7	26.1	103.90	This work
LCO/ Li_6PS_5Cl	Li-In		Li_6PS_5Cl	68	29	3.0−4.3	127	0	86.36	[1]
LCO/ Li_3YCl_6	Li-In	/	Li_3YCl_6	82.4	17.6	2.52−4.22	115	0	94.76	[2]
LCO/ Li_3InCl_6	Li-In	$Li_{10}GeP_2S_{12}$	Li_3InCl_6	70	30	2.5−4.2	127	0	88.90	[3]
LCO/ $Li_3Y(Br_3Cl_3)$	Li-In	/	$Li_3Y(Br_3Cl_3)$	60	37	2.62−4.27	124	0	74.4	[4]
LCO/ $Li_{1.75}ZrCl_{4.75}O_{0.5}$	Li-In	Li_6PS_5Cl	$Li_{1.75}ZrCl_{4.75}O_{0.5}$	75	25	2.5-4.2	137.5	0	103.13	[5]

(1) Kwak, H.; Han, D.; Lyoo, J.; Park, J.; Jung, S. H.; Han, Y.; Kwon, G.; Kim, H.; Hong, S.T.; Nam, K.W.; Jung, Y. S. New Cost-Effective Halide Solid Electrolytes for All-Solid-State Batteries: Mechanochemically Prepared Fe^{3+}-Substituted Li_2ZrCl_6. *Adv. Energy Mater.* **2021**, *11* (12), 2003190.

(2) Asano, T.; Sakai, A.; Ouchi, S.; Sakaida, M.; Miyazaki, A.; Hasegawa, S. Solid Halide Electrolytes with High Lithium-Ion Conductivity for Application in 4 V Class Bulk-Type All-Solid-State Batteries. *Adv. Mater.* **2018**, *30* (44), 1803075.

(3) Li, X.; Liang, J.; Luo, J.; Banis, M. N.; Wang, C.; Li, W.; Deng, S.; Yu, C.; Zhao, F.; Hu, Y.; Sham, T.K.; Zhang, L.; Zhao, S.; Lu, S.; Huang, H.; Li, R.; Adair, K. R.; Sun, X. Air-Stable Li_3InCl_6 Electrolyte with High Voltage Compatibility for All-Solid-State Batteries. *Energy Environ. Sci.* **2019**, *12* (9), 2665–2671.

(4) Liu, Z.; Ma, S.; Liu, J.; Xiong, S.; Ma, Y.; Chen, H. High Ionic Conductivity Achieved in $Li_3Y(Br_3Cl_3)$ Mixed Halide Solid Electrolyte via Promoted Diffusion Pathways and Enhanced Grain Boundary. *ACS Energy Lett.* **2021**, *6* (1), 298–304.

(5) Hu, L.; Wang, J.; Wang, K.; Gu, Z.; Xi, Z.; Li, H.; Chen, F.; Wang, Y.; Li, Z.; Ma, C. A Cost-Effective, Ionically Conductive and Compressible Oxychloride Solid-State Electrolyte for Stable All-Solid-State Lithium-Based Batteries. *Nat. Commun.* **2023**, *14* (1), 3807.
